# Agent based simulation with data driven parameterization for evaluation of social acceptance of a geothermal development: a case study in Tsuchiyu, Fukushima, Japan

**DOI:** 10.1038/s41598-022-07272-7

**Published:** 2022-02-28

**Authors:** Shuntaro Masuda, Kyle Bahr, Noriyoshi Tsuchiya, Tatsuya Takemori

**Affiliations:** Graduate School of Environmental Studies, Tohoku University, Sendai, Japan

**Keywords:** Environmental impact, Energy management

## Abstract

Development of geothermal power plants and local geothermal energy initiatives have lagged due to the social problems such as conflicts with stakeholders such as Onsen (Hot Spa) owners, despite the abundant geothermal resources. Study area was Tsuchiyu Onsen in Fukushima prefecture, Tohoku (Northeast) District, Japan, where the Great East Japan Earthquake and Fukushima Nuclear Accident occurred in 2011, and the reconstruction and local initiatives of geothermal energy were still unclear. Agent-based modeling is an effective methodology for modeling and analysis of opinion formation. Parameter estimation method was proposed to extract appropriate parameters from various factors through a Bayesian Network. The characteristics of stakeholders and communities that affected opinion formation in the survey area were successfully extracted. Here we show the sufficient methodology to quantify the characteristics of each person using survey data, and to extract the parameters of the agent by data-driven inverse analysis. By using this methodology, we could reproduce opinion diversity, which is a property of opinion formation in real communities. This result suggests that the model replicates the actual formation of opinion in Tsuchiyu, where the economy was boosted by the construction of a binary cycle power plant.

## Introduction

The March 2011 Great East Japan Earthquake, which had a magnitude of 9.0 and maximum seismic intensity of 7, caused great damage to infrastructure, including the collapse of buildings, and produced a tsunami over 39 m high that struck the Pacific coast of Japan^1^. As a result of the earthquake, 470,000 people were forced to evacuate their homes^2^. Within Fukushima Prefecture, 165,000 people were forced to evacuate and 37,000 of these have yet to return^3^. This fall in population has resulted in a decline in local industry.

Tsuchiyu-Onsen town (Tsuchiyu) in Fukushima City has been working on restoring its economy following the earthquake, focusing on a geothermal power project in an area of hot springs. Onsen (‘hot spa’ in English) are attractive to local people and tourists alike. The physical and reputational damage caused by the Fukushima Daiichi nuclear power plant accident has resulted in a 35% fall in the number of tourists visiting hot springs in the area, compared with the period before the earthquake^4^. Tsuchiyu is no exception to this trend, which has resulted in the closure of 5 of 16 hotels in the town^5^. To address this problem, a local volunteer-led organization that aims to assist the recovery of Tsuchiyu planned a geothermal project, and a 400 kW binary cycle power plant was installed in 2015^6^. A local company operates the power generation business and sells the electricity generated by the geothermal plant using the Feed-in Tariffs (FIT) law^7^, generating ~ 100 million yen per year, some of which is put towards urban development to attract tourists.

Although Tsuchiyu represents one of the most successful cases of geothermal development in a Japanese Onsen area, the total capacity of geothermal power plants has been largely unchanged for more than a decade, despite Japan’s vast geothermal potential, estimated at ~ 23 GW_e_^8,9^. This stagnation is due primarily to Japan’s reliance on nuclear power, which supplied about 31% of Japan’s electricity before the Fukushima Daiichi nuclear power plant accident^10^. Another factor is that hot spring owners operating hot spas are opposed to the development of geothermal power plants in their region^11,12^. As a result, although Japan has abundant geothermal resources, geothermal development has lagged behind in hot springs areas, and the use of hot springs has been limited to bathing.

Recent increases in the dissemination of information and public expectations of corporate social performance have meant public opinion is increasingly important in the success of resource development projects^13^. As such, obtaining social acceptance of a development has become the key to the effective utilization of geothermal resources. To better understand the formation of attitudes towards developers and opinions among stakeholders, and to evaluate policies encouraging the social acceptance of developments, a methodology is required for the quantitative analysis of opinion formation.

There are several kinds of studies on the quantitative analysis of opinion, but these consider ambiguous concepts that are difficult to measure. To quantitatively evaluate social acceptance, Boutilier and Thomson^14^ measured the attitude of stakeholders towards mining industries from surveys using questionnaires with 5-point ratings^14^. Statistical analysis of such empirical data provides a measure of the state of opinion that is close to the reality in society. However, Jensen and Chappin^15^ analyzed observations of concurrent events and noted that reaching a mechanistic understanding of social phenomena is more difficult than employing a statistical inference approach^15^. Although there are currently many unrelated methodologies employed to understand the characteristics of social acceptance, no standard practices are emerging^16–18^.

To analyze public opinion and consensus-building, many models of opinion formation have been developed using agent-based modeling (ABM) considering social interaction. In ABM, individual attributes are implemented as parameters of agents (i.e., people) to reproduce actual opinion formation. For instance, in the model of Deffuant et al.^19^, which is one of the most well-known, bounded confidence (i.e., the tolerance for others’ opinions) is used as a parameter^19^. Agents update their opinions by considering others’ opinions. If the difference in opinion between two agents who interact with each other exceeds their bounded confidences, they do not update their opinions.

This model reproduces agents’ behaviors by which they reconcile their opinions by cooperative operation, and their opinions diverge. In particular, the value of bounded confidence is a primary influence on agents’ behavior. Many other models employ a similar fundamental system of opinion formation; however, various kinds of agent parameters have been proposed to improve model performance^15,19–24^. This modeling methodology is suitable for understanding the behavioral trends of opinion formation, and for evaluating policies for consensus-building by scenario simulation.

There are some challenges, however, in applying the modeling methodology in analyzing opinion formation about attitudes towards geothermal developments in local communities. Duggins^24^ reported that one problem with previous models is that they have shown a lack of opinion diversity, which manifests in agents’ opinions converging to a single state (i.e., consensus) or to homogeneous groups (i.e., polarization)^24^. Mas (2014) proposed that these simulated behaviors are inconsistent with the high degree of persistent opinion diversity observed in many social settings^25^. It is possible that the simulation results reflect a lack of parameters and a model structure that does not appropriately reflect the characteristics of the agents.

Conventional models are useful in capturing the characteristics of groups in general terms; however, the characteristics of individuals need to be reflected in the model of opinion formation, because in local communities the behavior of an individual can have a large effect on the behavior of others. In addition to characterizing the individuals, the model should reproduce the diversity in opinions observed in the real world. To reflect real situations, the model should be designed to accept empirical data.

The main purpose of this paper is to develop a better model of opinion formation that has higher applicability and validity regarding opinion formation in local communities. To this end, data-driven parameterization is integrated with empirical data in an opinion formation model using ABM, which considers the characteristics and conditions of each object agent in the local community. ABM can describe behaviors of agents with mutual relations, which is not based on physical and mathematical functions, however, parameter between agents are determined simply and /or empirically. We proposed data driven parametarization and comparison with simple case. In addition, a new parameter estimation method is proposed that can extract appropriate parameters from various factors. This research is focused on stakeholders’ opinions about geothermal development. This analysis of social acceptability and social license is intended to contribute to the recovery of Tsuchiyu, a town recovering from a megaquake and related nuclear accident.

### Social survey

**Figure 1:**
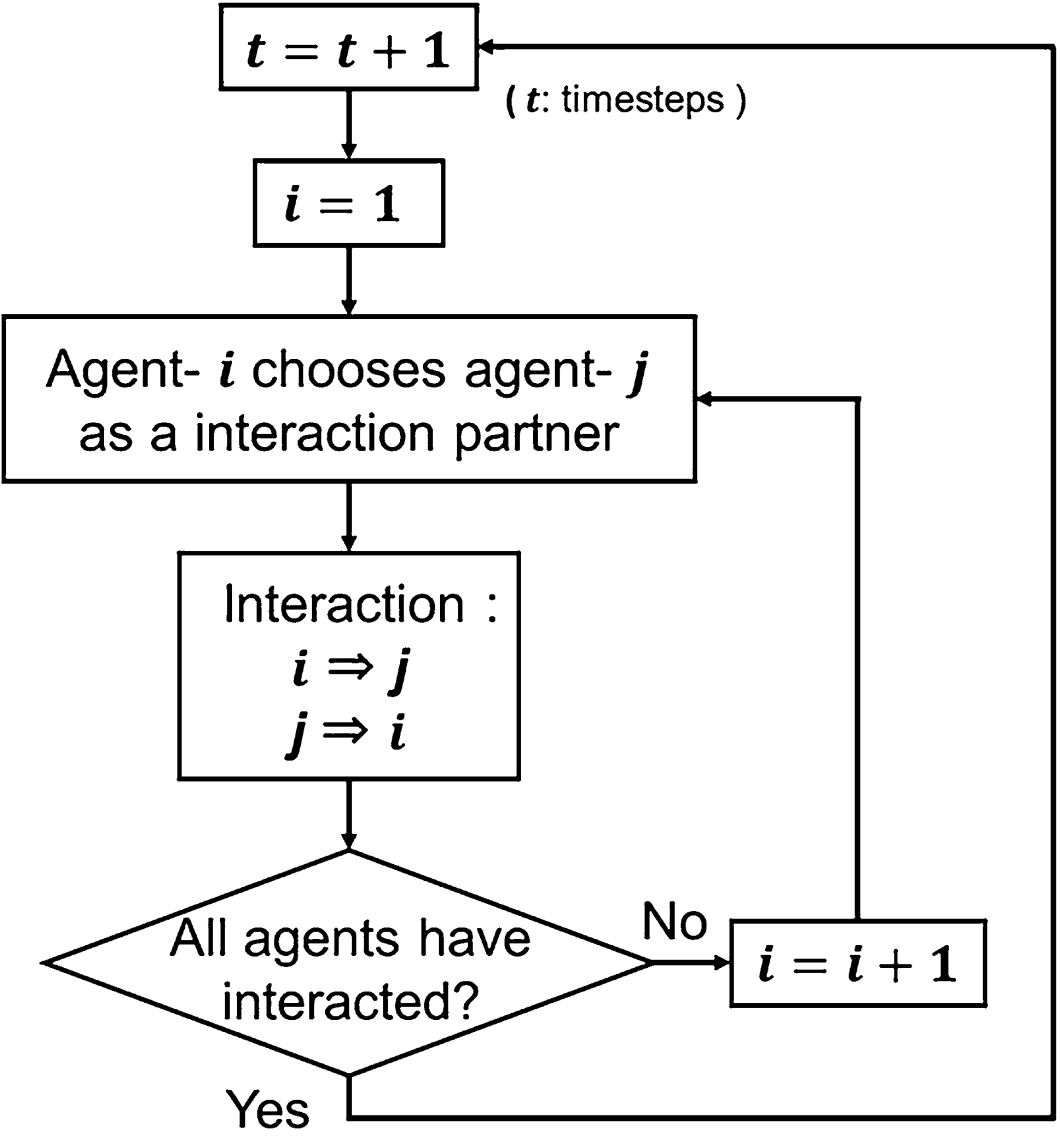
Flowchart of the opinion formation process in the ABM model employed in this study.

Agent-based modeling is a powerful simulation technique in exploring the dynamics of various kinds of phenomena using mathematical and computational methods^26–35^. For example, Arai and Terano^36^ proposed that the academic achievement gap in the social stratum is caused by the action rules for task achievement,i.e., by the motivation to learn^36^. In the medical field, Ishinishi et al.^37^ developed a model to identify when and where the spread of infection occurs by analyzing the simulation results^37^. In addition, ABM is not based on physical laws or their governing equations, and may be applied to the analysis of social surveys. ABM has been used to develop an opinion diffusion model based on the following steps: set the agents (i.e., people) as autonomous components; assign parameters to the agents; and set interaction rules that determine the partners of interaction and the degree to which the agents affect each other^38,39^. The opinion formation process employed by the model is shown in Fig. 1.

Figure 1 described opinion formation of stakeholder’s network as function of time and agents. Agent *i* interacts with agent *j* and then opinion between agents *i* and *j* are proceeded.

Most models employ the same fundamental mechanism; however, their behavior can be changed by modifying the parameters of the agents. In the model of Nakagawa and Bahr (2012)’ and Bahr and Nakagawa^19^, each agent is assigned parameters that describe influence and opinion^38,39^. In the model of Deffuant et al.^19^, agents have a tolerance parameter that is related to the acceptability of opinions from other agents^19^. A problem with previous models, however, is that they lack opinion diversity, which manifests in some models as the agents’ opinions converging to a single state (i.e., consensus) or to homogeneous groups (i.e., polarization)^24^.

In the present study, two main factors are identified that make these models less divergent than the real-world systems they attempt to simulate. The first factor is the appropriateness of parameters. Parameter setting in many models depends on the experience and observations of the modeler, which affects model behavior. Modelers chose factors as parameters that could affect opinion formation, such as age, social status, social position, and beliefs; however, there is no well-established method to determine the appropriate parameters for modeling a target system, and it is difficult to extract the critical or important factors that affect opinion formation in local communities. The second factor is the arbitrariness of parameter values, which are also determined based on the experience and observations of modelers. In many previous studies, parameter values have been assigned either uniformly or randomly to agents^24,39^.

While previous models have provided useful knowledge and made progress, data-driven parameterization is needed to assess the opinion dynamics in local communities in which the characteristics of each stakeholder have a significant impact on opinion formation. The approach taken in this study is ABM using data-driven parameterization and empirical data gathered through an interview survey.

To collect survey data, we interviewed people in Tsuchiyu-Onsen town (population ~ 400) regarding the social acceptance of geothermal development. The town can be roughly divided into the central, inner, and Tsuchiyu Pass areas. The central and inner areas are within ~ 1 km of each other, whereas the Tsuchiyu Pass area is about 20 km away. Tsuchiyu is a hot spring district with a long history, and is designated by the Ministry of the Environment (MOE) as a national recreational hot spring resort^5^. Tsuchiyu has traditionally used geothermal resources as a tourist attraction, which is the primary source of revenue. In 2011 the Great East Japan Earthquake caused damage to the town, and in 2015 the population decided to install a binary cycle power plant to support the revival of the area.

The questions about attitude were modified from Boutilier and Thomson^14^ (Supplementary Table 1). The subjects of the survey were 28 stakeholders in geothermal development, including private citizens, companies, organizations, Fukushima City Hall, and the Fukushima Prefectural Government. Tsuchiyu Onsen (Hot spring) town is small community, they have only 250 households. There were 16 hotels before Great East Japan Earthquake and Fukushima Nuclear Accident in 2011, but a number of hotels decreased into 11 and then one hotel was newly opened. In 2018, there were 12 hotels in Tsuchiyu Onsen. One hotel owner among them is outsider of Tsuchiyu. We surveyed 11 hotels in this study. 28 agents includes mostly all of hotel owners who are main agents of stakeholders in Onsen and geothermal area in Tsuchiyu.

The requirement for formal approval was waived by Research Compliance Promotion Office, Tohoku University, and the interview was conducted according to the Tohoku University Privacy Policy (see supplementary Table 1). Snowball sampling was used, which is a sampling method to find other subjects to interview through introductions by respondents^40^. Three types of data were collected from the survey: attribute data, data on opinion regarding geothermal development, and network data. The attribute data consist of 12 types of categorical data, such as age, gender, group association, Onsen well ownership, awareness of the environment, and knowledge of the benefits of geothermal development. The opinion data on geothermal development were gathered using a 5-point Likert scale. An option for a “Don’t Know” (DK) response was prepared and converted to an intermediate choice in the questionnaire responses, converting from DK to an intermediate choice had more than a trivial effect on the results of the analysis^41,42^. Although citizens of Tsuchiyu were connected through neighborhood associations and hot spring associations, there was no quantitative representation of the strength of the network connections; as such, the network data were developed from snowball sampling and the organizations to which people belonged.

## Results

### Parameter setting

From observations, interview surveys, and analysis of survey data, three factors were found to have strongly affected opinion formation in the area, as follows.

#### Attributes of stakeholders

The survey data show that attitudes of stakeholders depend on their attributes. For example, aggregate results of the questionnaire responses, divided by the form of Onsen ownership, show that stakeholders who have private Onsen wells are less likely to have positive opinions on geothermal development. There is a possibility, of course, that some other factors also affect their opinion formation.

#### Range of information sharing

The opinions of stakeholders depended on their locations. While stakeholders in different areas had different ideas and information about geothermal development and the power plant, stakeholders in the same area had quite similar ideas and information, indicating that information asymmetry is related to the geographical extent of information sharing.

#### Presence of influential stakeholders

There are several influential stakeholders in Tsuchiyu who in many cases hold important posts in primary local organizations and have great influence in the town. They have played central roles in consensus-building.

**Figure 2:**
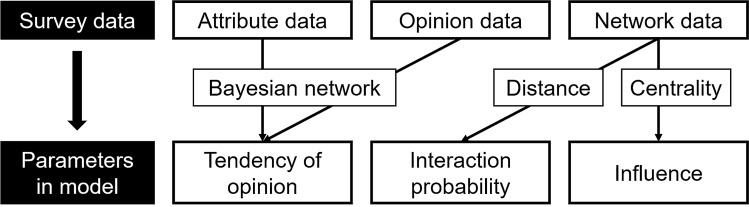
Parameter setting.

The three factors noted above, which are interpreted to strongly influence opinion formation, are integrated into the model of opinion formation as the following parameters: tendency of opinion (T), interaction probability (IP), and influence (I) (Fig. 2). The T parameter represents the bias of opinion direction (negative or positive), which depends on the attributes and characteristics of each stakeholder. This factor allows for consideration of the characteristics of each stakeholder, which may greatly improve the accuracy of the model of opinion formation in a local community. To reproduce the process of opinion diffusion and information asymmetry in the town, the IP parameter shows how frequently each stakeholder pair shares their opinion. Finally, the I parameter, which reflects the relative importance of each stakeholder on this specific issue, is included in the opinion formation model to reproduce the balance of power and social structure in the local community. The opinion formation model itself is based on mainstream models such as that of Deffuant et al.^19^.

The parameterization of T used a Bayesian network (BN), which is a probabilistic graphical model. Four types of attitudes and stakeholders’ attributes were set as variables in the BN, which acquired the causal relationships between attitudes and attributes.

Parameter T has a similar role to bounded confidence in the model of Deffuant et al.^19^, however, there are two key differences. First, T is extracted from various factors by probabilistic inference; therefore, it has the potential to reproduce more realistic decision making. Second, its values are defined as a probability distribution. In contrast, bounded confidence is a constant; therefore, fluctuation and error are not allowed. In the real world, even if human beings have strong tendencies, their decision making has some probabilistic fluctuations. Parameter T allows the extent of tendencies to be represented by the probability distribution.

**Figure 3:**
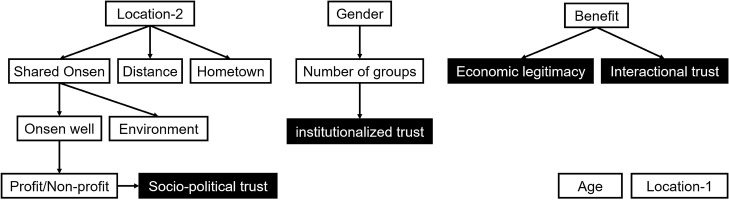
Bayesian network for the tendency of opinion in Tsuchiyu.

The four variables about opinion (opinion variables) have low, middle, and high choices, which are classified by the average score of 15 questions (Supplementary Table 2, 3). The result of the model learning is shown in Fig. 3, in which the opinion variables are indicated by black. Note that several constraints of the model structure are set to obtain an appropriate model. For example, the variable Gender was left unlinked because it is independent of all other variables. Also, opinion variables are set as target variables which have no out-links. The BN reveals the causal relationships among variables visually. The variables for profit/non-profit (the business form of their organizations), the number of groups (the number of groups in Tsuchiyu to which a stakeholder belongs), and benefit (the response to a question which asks the degree to which the respondent feels that the development is beneficial with respect to regional contributions) have direct causal relationships with opinion variables. Table 1 lists the conditional probability table (CPT) of the four opinion variables and the tendency of opinion of each agent based on attribute data.

**Table 1:** Conditional probability table of opinion variables.

	Benefit		
	Disagree	Neutral	Agree
**Economic legitimacy**			
High	0	0	0.64
Middle	0.50	1.0	0.36
Low	0.50	0	0
**Interactional trust**			
High	0	0	0.59
Middle	0	1.0	0.36
Low	1.0	0	0.05

	Profit		
	Profit	Non-profit	
**Socio-political trust**			
High	0.29	0.71	
Middle	0.71	0.21	
Low	0	0.07	

	Number of groups		
	Few	Middle	Many
**Institutionalized trust**			
High	0.43	0.17	0.67
Middle	0.57	0.83	0.11
Low	0	0	0.22

In previous models, some agents chose their interaction partners from within a network of their neighbors^19,43^, while others chose them in a random way^44^. The way in which partners are chosen, however, does not necessarily reflect the process of real social interaction. In fact, stakeholders in Tsuchiyu who are close tend to share their information and opinions preferentially. Therefore, geodesic distance among agents in social networks was adopted to weight the IP of their relationships with other agents as interaction partners. In addition, IP is defined as a parameter that is inversely proportional to distance, to take account of the exponential distance–decay effect (inverse square law) that is observed in natural and social phenomena.

**Figure 4:**
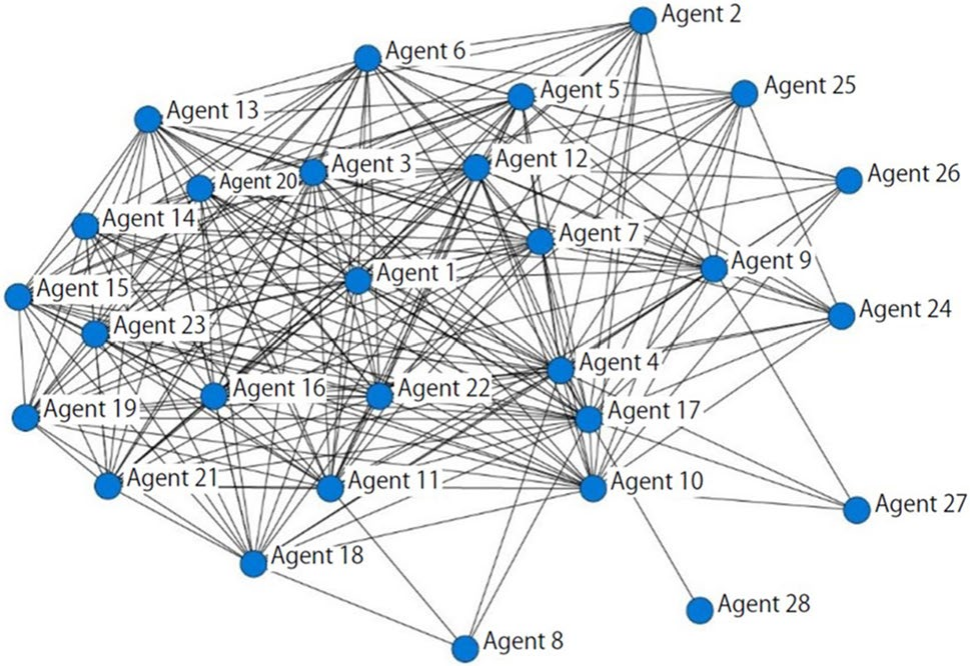
Stakeholder network in Tsuchiyu.

In this model, IP was calculated from the network data of 28 survey respondents (Fig. 4). The nodes are the respondents with their code names, and links represent direct relationships between respondents. The stakeholders with many links to others have higher eigenvector centrality, and come to the center. In the model simulations, the 28 agents choose interaction partners within this network depending on distances between them (Supplementary Figure 1).

Parameter I accounts for the importance of each agent in a social network, and Network centrality was used to quantify parameter I^44^. The index of stakeholders’ influence is PageRank, which was developed to evaluate the importance of Internet sites^45^. The decision to use PageRank was based on two points. First, PageRank is more appropriate to evaluate the influence of agents in a network compared with other centrality indexes because it considers the property and topology of the whole of the network. Second, while some centrality indexes require constraints in regard to network types (e.g., directed/undirected, connected/disconnected and weighted/unweighted), PageRank has broad applicability in terms of network type. The PageRank index was selected to develop a robust model that could be applied to various conditions and network data. This parameter is also calculated using the stakeholder network data shown in Fig. 4.

### Model validation

Three parameters were estimated from the survey data; however, two other variables, the scaling factors α (Eq. 1) and λ (Eq. 4), need to be determined. The value of the scaling factor determines the maximum value of opinion change during one time step in the simulation. If the value becomes too high, the opinion changes of agents also become so high that it leads to a non-equilibrium state of opinion formation or other unnatural behaviors. Conversely, too small a value influences the opinion distribution in transient states because of an unduly small opinion change during each time step. Factor λ determines the range of interaction of agents. It was not possible to estimate how far agents could interact with other agents in a real network by simply using the survey data. Factor λ can also change the speed of opinion diffusion and the transition behavior of opinion distribution. Therefore, the most appropriate set of values for α and λ needs to be determined.

A methodology is proposed to acquire the optimal parameter values by inverse analysis, to compare simulation data and survey data (target data) of the 28 stakeholders in Tsuchiyu. Inverse analysis is used for optimization problems in fields such as mathematics and engineering, and it fits simulation data to survey data by changing these parameter values and searching for the best fit values.

**Figure 5:**
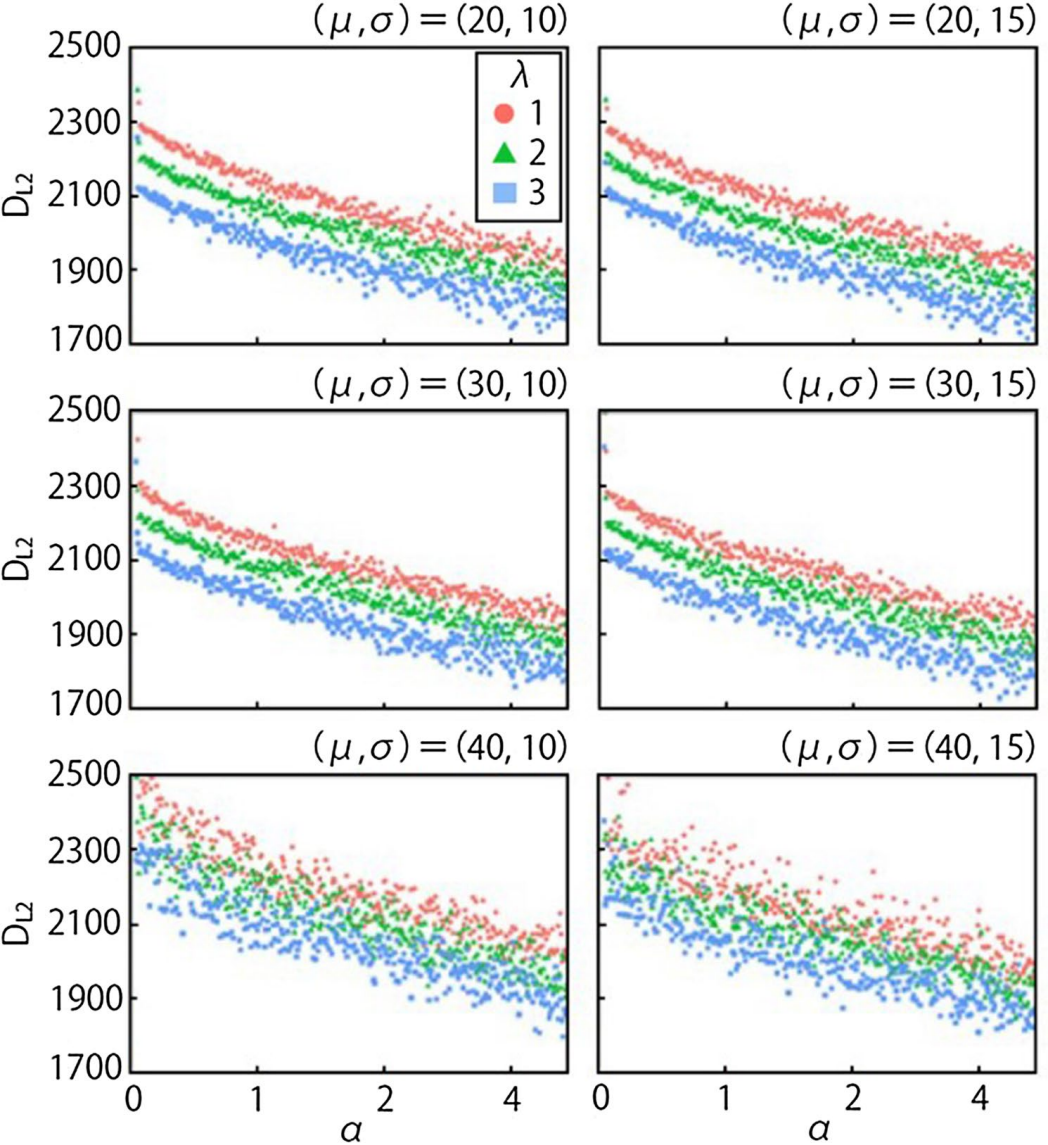
Relationships between L2-norm and the values of scaling factor α and λ. The horizontal axis is the value of α, the vertical axis is L2-norm (labeled D_L2)_, and the three plot colors represent the values of λ.

The results of inverse analysis of the initial opinion distributions with normal distribution with mean (i.e., center of opinion distribution) μ = 20, 30, 40 and standard deviation (i.e., extent of opinion distribution) σ = 10, 15 are shown in Fig. 5. To quantify the fitness of distributions, we set the difference of survey data and simulation data as an evaluation function, L2-norm (i.e., the Euclidean distance). The L2-norm means the square error between the two data. In all cases, L2-norm decreases as α and λ increase (Fig. 5), indicating that the fidelity of the model becomes better with higher values of α and λ. This is interpreted to mean that increasing these two factors makes the agents’ opinion distribution wider, a situation which is reflected in the Tsuchiyu data. In the proposed model, the degree to which agents exchange their opinion is proportional to the difference between their opinions (Eq. 1), so there is a strong cohesiveness to their opinion formation.

**Figure 6:**
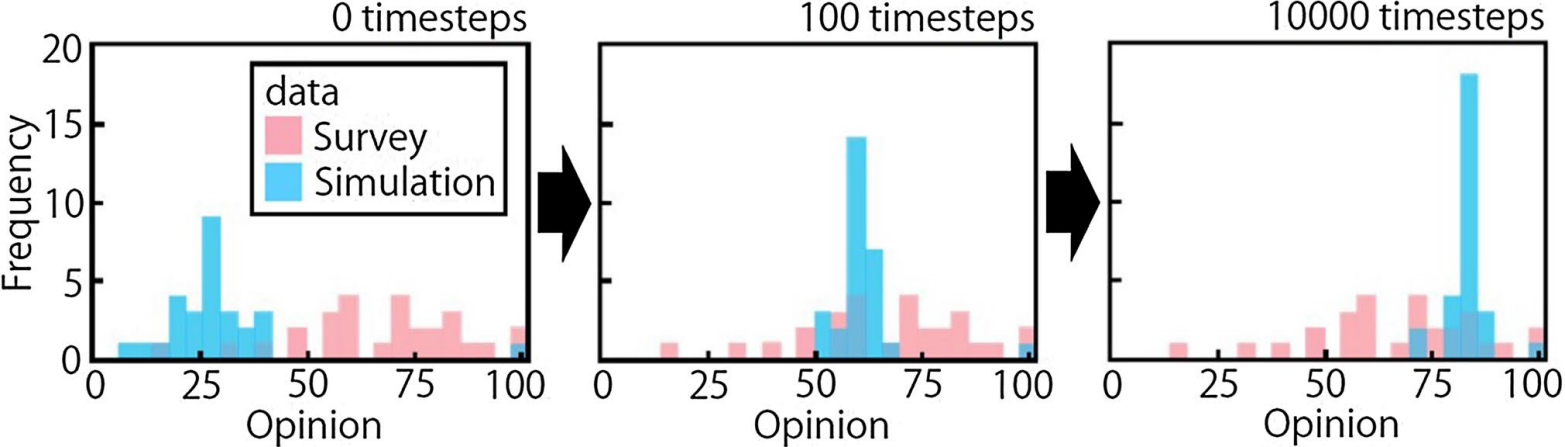
Comparison of survey data and simulation data for the opinion distribution of agents. The horizontal axis is opinion level (O), where 0 is negative and 100 is positive, and the graphs show the opinion distribution at 0, 100, and 1000 ts. The unit of the vertical axis, frequency, refers to the number of agents. Blue and red represent simulation and survey data, respectively.

Figure 6 shows the opinion distributions of the survey data and simulation data with α = 3.45, and λ = 3. Note that the survey data were only collected once, and so the distribution of the survey data is the same in all three panels. The majority of people come to have positive opinions (movement of data to the right) under the influence of an opinion leader (OL) who maintains a positive opinion of the development. In the transition of opinion, a state is reached that is closest to the survey data. For instance, the mean of the opinion distribution at 100 timestep (ts) is more similar to the survey data than that at 0 ts.

In the example of the model for opinion formation in Tsuchiyu (Fig. 6), opinions are aggregated as time goes on, and most agents come to have similar opinions around O = 80 at 1000 ts. The center of the distribution approaches that of the survey data over time; however, the shape of the distribution of survey data is flatter than that of the simulation data. This difference directly affects L2-norm, which represents the sum of the squared error of the two cumulative distribution functions. In other words, L2-norm becomes smaller when the opinion distribution by the model becomes flatter. The scaling factor α adjusts the scale of opinion change for all agents in one interaction. Then, if the value of α increases, the opinion change of agents also increases, which is interpreted to mean that the higher α value made it more difficult for their opinions to converge; consequently, L2-norm decreased. In terms of λ, the higher λ value means that agents are more likely to interact with those agents who are located close to them in a network, thereby preventing information transmission in the network and limiting opinion convergence, which could also lead to a smaller L2-norm. The optimal parameter values obtained by inverse analysis of the survey data are α = 3.45 and λ = 3, which are used in the subsequent simulations.

In this research, agent parameters were extracted from the survey data. To verify the impact of the introduction of the parameters that reflect individual characteristics, the simulation with T, which plays an important role in our model, was compared with the simulation without T. The simulations were conducted with survey data for the 28 stakeholders in Tsuchiyu. A local geothermal industry was set as an OL. Initial opinions were assigned to agents randomly using a normal distribution of μ = 50 and σ = 10, which means that the majority has a neutral opinion at 0 ts. With these conditions, a steady state and a transition process was observed in which an OL leads other agents to a positive opinion. To exclude the influences of stochastic fluctuations in the simulations, 100 simulations were conducted for each condition and these results were integrated. As the resulting histograms did not represent the results well, a kernel density estimation was used, which is a non-parametric method to estimate the probability density function of stochastic variables. The density function in the stats package in R (a programming language for statistical analysis) was used to obtain the subsequent opinion distribution of simulation results. The kernel function was set as Gaussian, and bandwidth was set to 1.

**Figure 7:**
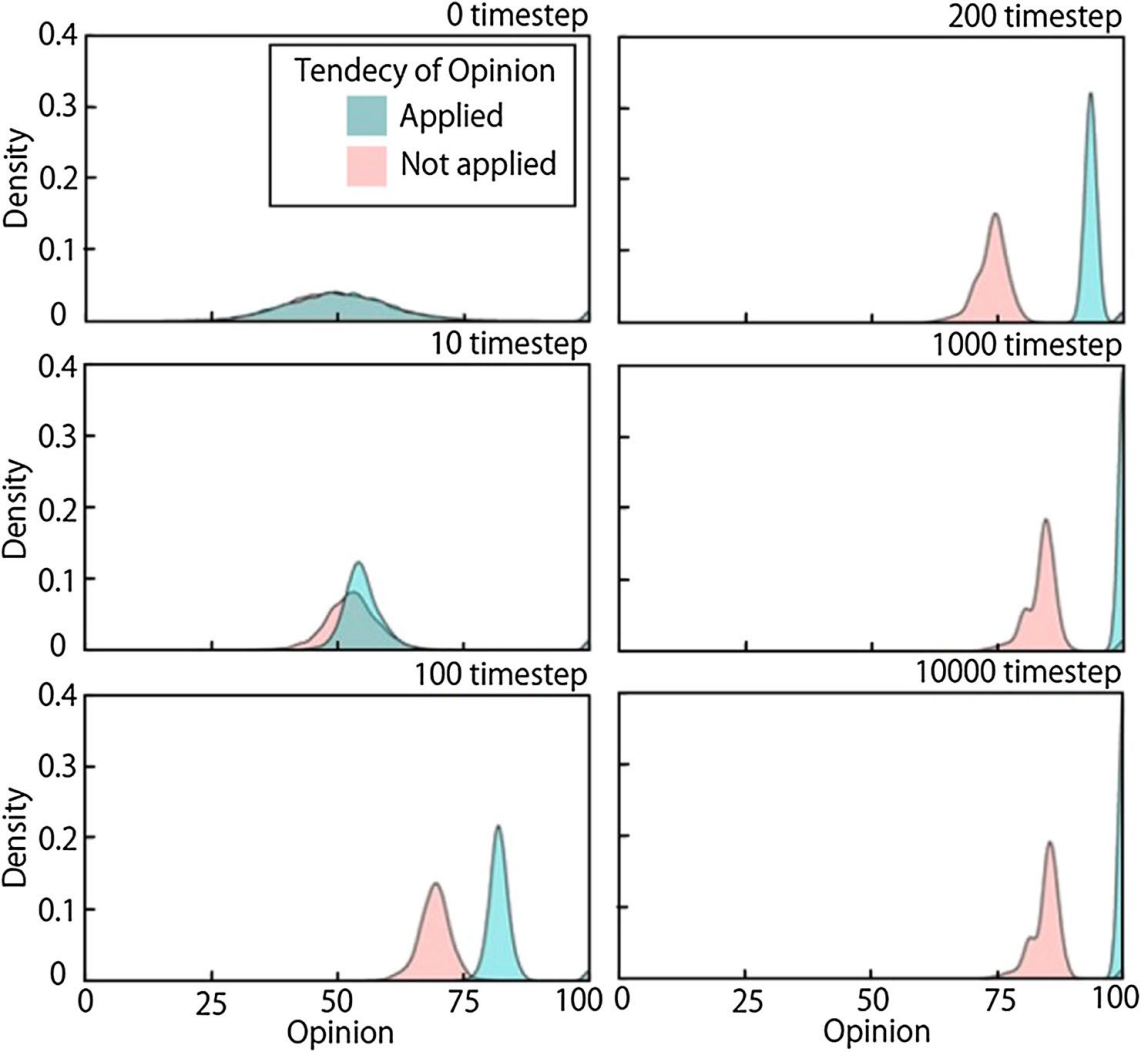
Comparison of opinion distributions with and without T at 0, 10, 100, 200, 1,000, and 10,000 ts.

Figure 7 compares the opinion distributions with T (red curve) and without T (blue curve) at 0, 10, 100, 200, 1000, and 10,000 ts. At 0 ts, opinions follow a normal distribution with a central focus on neutral (O = 50), except for an OL with O = 100.

At 10 ts, opinions are gradually changed and the data are more clustered. The speed of aggregation with T is slower than that without T, because it becomes difficult for agents to accept opinions from others due to the tendency of opinion, and the opinion cohesion of the entire group is slower to develop. At 10,000 ts, enough time has passed for opinion forming in the network, and all agents have the same positive opinion (O = 100) without T applied, while agents with T applied do not exceed a certain level of positive opinion (75–100). As a result, when a tendency of opinion, which reflects the characteristics of individual stakeholders, was not applied to the model, agents reached a complete consensus; Duggins^24^ mentioned this typical behavior in previous models^24^. On the other hand, when T was applied, the result was opinion diversity, which is observed in the real world. This indicates that the introduction of characteristics of individual agents from survey data and data-driven parameterization could reproduce opinion diversity, which is a property of opinion formation in real communities.

## Discussion

Tsuchiyu is located in the southwest part of Fukushima City, on a plateau at an elevation of 450 m above sea level, surrounded by the Azuma Mountain Range. Although it is located relatively close to the center of Fukushima City (about 16 km away), it is a resort area with a rich natural environment. In addition to being designated a national hot spring resort by the MOE, the Arakawa River flowing through Tsuchiyu has the cleanest river water in Japan. Tsuchiyu is also famous for its kokeshi dolls, a traditional handicraft, and is one of the most famous kokeshi doll towns in the Tohoku region. The central part of Tsuchiyu is clustered in a small area, with more than 10 hot spring inns at the center, as well as restaurants and souvenir shops. In addition, there are six satellite Onsen located 20 km from the main area. Although the Onsen are distant from each other, they are linked by networks such as a hot spring association.

In recent years, however, the population of Tsuchiyu has been declining and aging. With a population of about 300 people (about 170 households) and an aging rate (Percentage of population aged 65 and over) of about 56%, the village has become marginal village, which is difficult to maintain their social symbiosis because of the depopulation and aging.

The Great East Japan Earthquake, which occurred in March 2011, resulted in the closure of five inns, and the capacity of the hot spring resort to accommodate guests was reduced by half. As a result, the number of tourists dropped off significantly, and the survival of the town was at stake. To help Tsuchiyu recover from this critical situation, a community development company was established with local capital, led by the local leader. This leader then led the way to the installation of a 400 kW binary cycle power plant in 2015. The leader had such influence that his opinions become the policy for the entire region, and a plan for regional development using the proceeds from the sale of electricity from the binary cycle power plant was quickly approved. From this point of view, he can be called an opinion leader of the region.

To assist the recovery of Tsuchiyu, which was struck by a megaquake and a nuclear accident, an opinion formation model was developed that has high applicability and validity for opinion formation among stakeholders in local communities. The results of the simulation are compared from three perspectives: network distance, human characteristics, and time, to assess if a quantitative model is established.

First, although Tsuchiyu is divided into three parts, the respondents actually belong to the same community and form a dense network. To duplicate these characteristics in the network data, a survey was conducted using snowball sampling, clarifying the organizations to which the survey respondents belonged. By generating these network data in the simulation, it was possible to simulate the realistic selection of opinion exchange partners. Second, the human characteristics of Tsuchiyu’s residents were confirmed in the survey, which showed that the stakeholders have different attributes and opinions. From the survey results, it was possible to reproduce the probabilistic exchange of opinions through the introduction of BN. Third, the time factor leading up to the installation of a binary cycle power plant in the aftermath of the Great East Japan Earthquake was strongly influenced by an opinion leader in the local organization. This situation was replicated by setting an opinion leader (OL) who maintains his positive opinion in the simulation.

Based on the comparison of simulation results, the model is judged to provide a quantitative assessment of the local network, which was previously perceived to be qualitative. In addition, opinion diversity could be reproduced in simulations. This suggests that a certain level of evaluation is possible through a survey using snowball sampling with some consideration of attributes.

To further improve the approach, it would be better to increase the sample size of the survey or to smooth the probability distributions in Table 1. The parameter T depends on the sample size. Although the survey in this simulation had 28 respondents, parts of the probability distribution are clear. For instance, in Table 1 for an agent whose benefit variable is disagree, his tendency for interactional trust is negative at a probability of 100%. This means that he never accepts positive or neutral opinions about interactional trust, which is unrealistic behavior. In the simulation, however, this error agent generated agents who have a strong bias towards a negative opinion, kept relatively neutral opinions, and contributed to opinion diversity.

In this model, the opinions of agents approach each other (Eq. 1) and finally converge to a consensus if there are no special conditions, such as the presence of agents who maintain their opinion. Tendency of opinion represents bias towards opinions from others. Consequently, if this parameter adopts a concept of repulsion (i.e., agents who update their opinions contrary to their interaction partners)^43^, the model could extend the range of its expression and could reproduce more realistic behaviors. Inverse analysis enables comparison of multiple proposed models and the selection of a model with the highest validity.

Another problem with the proposed model, is that the time series data for various situations were not applied. In the future, the performance and validity of the model might be improved by introducing the time series data. Applying the model to other regions should also be considered.

## Methods

### Proposed model

The flowchart of opinion formation process in the model is represented in Fig. 1. The process is roughly divided into two parts. At first, agents (stakeholders) choose their interaction partners. Next, agents exchange their opinions with the partners, and update their opinions. By repeating the series of actions, the model reproduces opinion formation in a community. In this model, agents choose interaction partners with interaction probability, and update their opinions considering opinions and parameter I of partners and their own parameter T. Take the interaction agent-*j* → agent-*i* (i.e., agent-*j* tells his opinion to agent-*j*) as an example to be explained. In this case, agent-*i* updates his opinion based on the following equation:

1 $${\left. {O_{i} } \right|_{t + 1} = \left. {O_{i} } \right|_{t} + \alpha \cdot I_{j} \left( {\left. {O_{j} } \right|_{t} - \left. {O_{i} } \right|_{t} } \right)T_{i} ,}$$

where $\left. {O_{i} } \right|_{t}$ is the opinion at *t* ts ($0 \le O_{i} \le 100$); *α* is the scaling factor, which is a positive real number; $I_{j}$ is the influence of agent-*j* ($0 \le I_{j} \le 1$); and $T_{i}$ is a function that varies between 0 and 1 by the probability distribution based on the tendency of opinion of agent-*i*. The method of determining the value of $T_{i}$ is shown below. The value of $O_{i}$ is classified into three opinion categories: negative ($0 \le O_{i} < 33.3$), neutral ($33.3 \le O_{i} \le 66.7$), and positive ($66.7 < O_{i} \le 100$). For instance, if agent-*j* tries to relate his positive opinion ($66.7 < \left. {O_{j} } \right|_{t} \le 100$) and agent-*i* has a tendency of opinion towards negative (i.e., the probability distribution is biased towards negative), $T_{i}$ is likely to become zero. Then, the second term in Eq. 1 becomes zero, and agent-*i* does not accept the opinion from agent-*j* and does not update his opinion.

### Parameterization

#### Tendency of opinion

To parameterize T, a BN was used, which is a probabilistic graphical model. In a BN, stochastic variables and their causal connections are represented by nodes and arrows^46^. The causal relationships between variables that have direct connections are represented as a CPT. A BN can be applied to discrete variables, and it is a great advantage for parameterization in this research because the survey data include categorical data that have many constraints for multivariate analysis. To acquire the causal relationships in a CPT, T can be obtained as a probability distribution. In the proposed model, agents decide whether they accept opinions from interaction partners based on T.

A BN is acquired with machine learning and probabilistic inference algorithms by exploring the optimal combination of all variables. Inferences form the BN model were assessed using score-based algorithms that are heuristic optimization algorithms and rank model structures with respect to goodness-of-fit^47,48^. This method was adopted because requires completely directed graphs, which is a complete graph with a directed edge between any two vertices, that can calculate all CPTs in the models. Among the possible algorithms, the hill climbing method was used, which is a standard optimization technique for locating local maxima and minima. Bayesian information criterion (BIC) scores were used for the learning algorithms. For implementation of the BNs, the bnlearn package was used in R, which is a programming language for statistical analysis^47^.

#### Interaction probability

The probability that agent-*i* chooses agent-*j* as an interaction partner is represented as follows:

2 $${IP_{ij} \propto \frac{1}{{\text{D}_{ij}^{\lambda } }},}$$

3 $${\sum \limits_{k = 1}^{n} IP_{ik} = 1,}$$

where ${\text{D}}_{ij}$ is the geodesic distance between agent-*i* and agent-*j* in a network, λ is the extent of exponential distance-decay effect that is applied to all agents, and *n* is the number of agents. Note that the sum of the set of *IP* for agent-*i* ($IP_{{\varvec{i}}}$) becomes 1 because it is a probability distribution. Then, $IP_{ij}$ is acquired to be normalized and is represented as follows:

4 $${IP_{ij} = \frac{1}{{\text{D}_{ij}^{\lambda } }} \cdot \frac{1}{{\sum \nolimits_{k = 1}^{n} \frac{1}{{D_{ik}^{\lambda } }}}}}$$

The equation shows that agents in a community with low λ values tend to interact with agents at greater distance than agents with high λ values. Therefore, the λ value resembles the characteristic of interaction in a community, whether it is introverted or extroverted. The interaction probability $IP_{ij}$ is calculated form the adjacency matrix of the social network.

#### Influence

The PageRank of node-*i*, $C_{pag} \left( i \right)$, is given as

5 $${C_{pag} \left( i \right) = d\sum \limits_{j} \frac{{C_{pag} \left( j \right)}}{{L_{j} }} + \frac{1 - d}{n}}$$

where *d* is a damping factor (in a range between 0 and 1, generally 0.85) and $L_{j}$ is the number of links to other nodes of node-*j*. There are two important points to consider in using PageRank rather than eigenvector centrality. At first, each node is given the value $\left( {1 - d} \right)/n$ equally. This means that transition probabilities (adjacency matrix) are subtracted by the rate of a damping factor, and are then distributed to all nodes. In other words, in eigenvector centrality, the transition probability of node-*i* to node-*j* is 0 or 1 (connected or disconnected); however, in PageRank, it becomes 0.15 or 0.85. The subtracted value $\left( {1 - d} \right) = 0.15$ is distributed equally to all nodes. This enables disconnected graphs and directed graphs to obtain positive and real eigenvalues. The second important point is that the centralities received from other nodes ($C_{pag} \left( j \right)$) are divided by the number of their degree ($L_{j}$). This is because the in-link from a node that has fewer links is more highly evaluated because of its importance and scarcity in PageRank. This is considered an advantage over eigenvector centrality.

There were two main reasons for selecting PageRank. First, compared with other centrality indexes, PageRank is more appropriate in evaluating the influence of agents in a network because it considers the property and topology of the whole network. Second, while some centrality indexes are limited in terms of network type (i.e., directed/undirected, connected/disconnected and weighted/unweighted), PageRank has broad applicability in terms of the type of network data accepted.

### Model validation

To acquire the most appropriate set of values for the scaling factors α (Eq. 1) and λ (Eq. 4), inverse analysis was used to compare simulation data and survey data (target data) from the 28 stakeholders in Tsuchiyu.

Initial values of λ were set to 1, 2, and 3, which were assumed, based on well-known natural laws of distance-decay such as inverse square laws and other studies^49^. The range of values of α was based on the model of Bahr^44^. In this model, the maximum value of opinion change is fixed to 20% of the range of opinion. In our model, the maximum value of opinion change can change with the maximum value of influence (*I* of agents; therefore, the value was calculated from the maximum value of *I*. From Eq. 1, the maximum value of *α* is represented as follows:

6 $${\max \left( \alpha \right) = \frac{20}{{\max \left( {I_{j} } \right) \cdot 100 \cdot 1}} = \frac{1}{{5 \cdot \max \left( {I_{j} } \right)}}.}$$

In the Tsuchiyu case, $\max \left( {I_{j} } \right)$ is 0.0580 and $\max \left( \alpha \right)$ about 3.45. Thus, the range of the value of α was determined as $0 < {\upalpha } \le 3.45$.

The initial settings for the inverse analysis were three parameters for 28 agents, values of two variable parameters λ and α as experimental conditions, and the initial opinion distribution of agents. Next, simulations were conducted by changing the two values, and exploring the set of values with which the opinion distribution in a transient or steady state approaches the closest to the opinion distribution of the survey data. It was assumed that the majority of respondents in Tsuchiyu had negative opinions because the residents have refused geothermal developments in the past; therefore, negative opinions were distributed to agents randomly. In the model, the range of negative opinion was from 0 to 50, and therefore the opinions had a normal distribution with mean (i.e., center of opinion distribution) $\mu = 20, 30,40$ and standard deviation (i.e., extent of opinion distribution) $\sigma = 10, 15$. Several distributions were set to observe and avoid the effect of an initial opinion distribution on the final distribution. To reproduce the consensus-building in Tsuchiyu, a local geothermal industry leader, who has led other stakeholders to a positive opinion, was set as an opinion leader (OL) who does not change his positive opinion ($O_{{{\text{OL}}}} = 100$).

To quantify the fitness of distributions, their difference was set as an evaluation function L2-norm. L2-norm was chosen because it is widely used in engineering and science, and is well known as an Euclidean distance. The L2-norm represents the square error between two functions, *p* and *q*:

7 $${D_{L2} \left( {p,q} \right) = \int \limits_{ - \infty }^{\infty } \left( {p - q} \right)^{2} dx}$$

In this case, *p* and *q* are survey data and simulation data, respectively. The difference between the data is well represented by L2-norm, and its simple calculation reduces the computational load of inverse analysis, so it was adopted as the evaluation function for inverse analysis. The above equation was applied to cumulative opinion distribution functions of survey data and simulation data (Fig. 6). The minimum values of the evaluation function ensure that the most appropriate values for combinations of λ and α were used in the simulation.

## Supplementary Information

Supplementary Information.

## Data Availability

Questionnaire and results of the social survey and the input data for the model is available via the figshare repository at https://doi.org/10.6084/m9.figshare.14545929.v1.

## Code availability

Input data and the NetLogo code for the model is available via the figshare repository at https://doi.org/10.6084/m9.figshare.14617029.v1.
