# Supplementary material for: Agent based simulation with data driven parameterization for evaluation of social acceptance of a geothermal development: a case study in Tsuchiyu, Fukushima, Japan
Source: Sci Rep. 2022 Feb 28;12:3314. doi: 10.1038/s41598-022-07272-7 (PMC8885700; doi:10.1038/s41598-022-07272-7)
Supplement: Supplementary file 1 — Supplementary Information. [file 41598_2022_7272_MOESM1_ESM.docx]

**Supplementary Information for Agent Based Simulation with Data Driven Parameterization for Evaluation of Social Acceptance of a Geothermal Development: A case study in Tsuchiyu, Fukushima, Japan**

**Shuntaro Masuda^1^, Kyle Bahr^1^, Noriyoshi Tsuchiya^1^*, Tatsuya Takemori^1^**

1 Graduate School of Environment, Tohoku University, Sendai, Japan

*Corresponding Author

Supplementary Table 1 Questions about perception of the geothermal industry and development.

| **Questions** | |
| --- | --- |
|  | We are very satisfied with our relationship with the geothermal industry. |
|  | My (community / organization) and geothermal industry have a similar vision for the future development of this region. |
|  | We must have the cooperation of geothermal industry to achieve our most important goals. |
|  | The geothermal industry listens to our opinions. |
|  | The geothermal industry does what it says regarding our (community/organization). |
|  | The geothermal industry openly shares information related to us. |
|  | We can gain from a relationship with geothermal industry. |
|  | In the long run geothermal industry makes a contribution to the welfare of the entire region. |
|  | The geothermal industry gives more help to those who are most affected. |
|  | The presence of geothermal industry is a benefit for us. |
|  | The geothermal industry takes our interests into account. |
|  | The geothermal industry shares decision making with us on matters of interest to my (community / organization). |
|  | The geothermal industry is fair to all. |
|  | The geothermal industry respects our way of life. |
|  | The geothermal industry cares about our interests. |

Supplementary Table 1 Continued.

| **Questions** | |
| --- | --- |
|  | The people of our (organization / community) speak well of the geothermal industry. |
|  | What do they say? (Free answer) |
|  | The geothermal industry protects its own interests in hot springs. |
|  | Geothermal development does not adversely affect the local environment, including water, soil, and air. |
|  | Geothermal energy is more viable than solar energy. |
|  | Geothermal energy is more viable than wind energy. |
|  | Geothermal energy is a public resource for our community. |
|  | We are satisfied with the rate of progress of geothermal development in our community. |
|  | We can gain by proceeding with geothermal development in this area. |
|  | Based on your current understanding, what is the most appropriate / realistic strategy for developing the geothermal resource in your community. (Choose top 3 from 10 choices, “bathing and swimming,” “structure heating,” “snow management,” “greenhouse,” “fish farming” “industrial food/lumber drying,” “mineral leaching,” “electric power generation,” “don’t support geothermal,” “other.”) |
|  | Please explain your choices. (Free answer) |

Interview was conducted according to Tohoku University Privacy Policy (<https://www.tohoku.ac.jp/japanese/site/siteinfo/01/siteinfo0103/>, *in* *Japanese*).

Supplementary Table 2 Dataset for construction of a Bayesian network.

| **Stakeholder** | **Attributes and characteristics** | | | | | |
| --- | --- | --- | --- | --- | --- | --- |
|  | **Gender** | **Age** | **Shared onsen** | **Onsen well** | **Profit / Non-profit** | **Number of groups** |
| HIS-1 | Male | 60 | Yes | Yes | Non-profit | Many |
| NAU | Male | 60 | No | No | Non-profit | Middle |
| NTC-1 | Male | 60 | Yes | Yes | Non-profit | Many |
| RDC | Male | 60 | No | No | Non-profit | Many |
| NTC-4-1 | Male | 50 | No | No | Non-profit | Middle |
| LS | Female | 50 | Yes | Yes | Non-profit | Middle |
| HIS-4 | Male | ≤40 | Yes | No | Non-profit | Middle |
| FB-2 | Female | 50 | No | No | Non-profit | Few |
| IGT-1 | Male | 60 | No | No | Profit | Many |
| IGT-2-1 | Female | ≤40 | No | No | Profit | Few |
| WA | Female | 60 | No | No | Non-profit | Middle |
| HIS-2 | Male | ≤40 | Yes | No | Non-profit | Middle |
| LBM | Male | 70 | No | No | Profit | Many |
| HOKT | Male | 60 | Yes | No | Profit | Many |
| HOKW | Male | ≤40 | Yes | Yes | Profit | Middle |
| HONE-3 | Male | 50 | Yes | Yes | Profit | Middle |
| TA-3 | Male | 50 | No | No | Non-profit | Many |
| LBN | Male | 70 | No | No | Profit | Middle |
| HONI | Male | 70 | Yes | Yes | Profit | Middle |
| HOAK | Male | 70 | Yes | Yes | Profit | Many |
| HOSG-2 | Male | ≤40 | Yes | Yes | Profit | Middle |
| HOYS-2 | Male | ≤40 | Yes | Yes | Profit | Many |
| HOST | Male | 60 | Yes | No | Profit | Middle |
| FCTO | - | - | No | No | Non-profit | Few |
| FCED | - | - | No | No | Non-profit | Few |
| FPED | - | - | No | No | Non-profit | Few |
| JE | - | - | No | No | Profit | Few |
| INC | - | - | No | No | Profit | Few |

Supplementary Table 2 Continued.

| **Stakeholder** | **Attributes and characteristics** | | | | |
| --- | --- | --- | --- | --- | --- |
|  | **Location-1** | **Location-2** | **Distance** | **Hometown** | **Environment** |
| HIS-1 | Fukushima | Central area | Close | Tsuchiyu | Disagree |
| NAU | Fukushima | Central area | Close | Tsuchiyu | Agree |
| NTC-1 | Fukushima | Central area | Close | Tsuchiyu | Disagree |
| RDC | Fukushima | Central area | Close | Tsuchiyu | Agree |
| NTC-4-1 | Fukushima | Central area | Close | Tsuchiyu | Agree |
| LS | Fukushima | Central area | Close | Other | Agree |
| HIS-4 | Fukushima | Central area | Close | Tsuchiyu | Agree |
| FB-2 | Fukushima | Central area | Close | Tsuchiyu | Agree |
| IGT-1 | Fukushima | Central area | Close | Tsuchiyu | Agree |
| IGT-2-1 | Fukushima | Central area | Close | Other | Neutral |
| WA | Fukushima | Central area | Close | Other | Agree |
| HIS-2 | Fukushima | Central area | Close | Tsuchiyu | Agree |
| LBM | Fukushima | Central area | Close | Tsuchiyu | Agree |
| HOKT | Fukushima | Inner area | Very close | Tsuchiyu | Disagree |
| HOKW | Fukushima | Inner area | Very close | Other | Neutral |
| HONE-3 | Fukushima | Central area | Close | Other | Agree |
| TA-3 | Fukushima | Central area | Close | Other | Agree |
| LBN | Fukushima | Central area | Close | Tsuchiyu | Neutral |
| HONI | Fukushima | Central area | Close | Tsuchiyu | Neutral |
| HOAK | Fukushima | Tsuchiyu pass | Middle | Tsuchiyu | Neutral |
| HOSG-2 | Fukushima | Tsuchiyu pass | Middle | Tsuchiyu | Disagree |
| HOYS-2 | Fukushima | Tsuchiyu pass | Middle | Tsuchiyu | Disagree |
| HOST | Fukushima | Central area | Close | Other | Agree |
| FCTO | Fukushima | Outside | Far | Other | Neutral |
| FCED | Fukushima | Outside | Far | Other | Agree |
| FPED | Fukushima | Outside | Far | Other | Neutral |
| JE | Other | Outside | Very far | Other | Neutral |
| INC | Tsuchiyu | Outside | Very far | Other | Neutral |

Supplementary Table 2 Continued.

| **Stakeholder** | **Attributes and characteristics** | | | | |
| --- | --- | --- | --- | --- | --- |
|  | **Benefit** | **Economic legitimacy** | **Interactional trust** | **Socio-political trust** | **Institutionalized trust** |
| HIS-1 | Disagree | Low | Low | Low | Low |
| NAU | Agree | Middle | Middle | Middle | Middle |
| NTC-1 | Agree | High | High | High | High |
| RDC | Agree | High | High | High | High |
| NTC-4-1 | Neutral | Middle | Middle | High | Middle |
| LS | Agree | High | High | High | Middle |
| HIS-4 | Agree | High | Middle | High | Middle |
| FB-2 | Agree | Middle | Middle | High | High |
| IGT-1 | Agree | High | Middle | High | High |
| IGT-2-1 | Agree | Middle | Middle | Middle | Middle |
| WA | Agree | Middle | High | Middle | Middle |
| HIS-2 | Agree | High | High | High | Middle |
| LBM | Agree | High | High | High | High |
| HOKT | Agree | High | High | Middle | High |
| HOKW | Neutral | Middle | Middle | Middle | Middle |
| HONE-3 | Agree | High | High | High | High |
| TA-3 | Agree | High | High | High | High |
| LBN | Agree | Middle | High | High | Middle |
| HONI | Agree | High | Middle | Middle | Middle |
| HOAK | Agree | Middle | Low | Middle | Low |
| HOSG-2 | Disagree | Middle | Low | Middle | Middle |
| HOYS-2 | Neutral | Middle | Middle | Middle | Middle |
| HOST | Agree | High | High | Middle | High |
| FCTO | Agree | Middle | High | High | High |
| FCED | Agree | High | High | High | High |
| FPED | Agree | Middle | Middle | Middle | Middle |
| JE | Neutral | Middle | Middle | Middle | Middle |
| INC | Agree | High | Middle | Middle | Middle |

Supplementary Table 3 Explanation of variables in the dataset.

| **Variable** | **Explanation** | **Choice** |
| --- | --- | --- |
| Gender | Gender of respondents. We couldn’t collect data of external stakeholders. | ・Male  ・Female |
| Age | Their age. We couldn’t collect data of external stakeholders. | ・≤40 (including 20-40’s)  ・50 (50’s)  ・60 (60’s)  ・70 (70’s) |
| Shared onsen | Whether they possess onsen supplied by shared onsen source or not. | ・Yes  ・No |
| Onsen well | Whether they possess private onsen source or not. | ・Yes  ・No |
| Location-1 | The location of their organization in Japan. | ・Fukushima  ・Tohoku (except Fukushima) |
| Location-2 | The location of their organization in Tsuchiyu. | ・Central area  ・Inner area  ・Tsuchiyu pass  ・Other |
| Distance | The distance between the location of the base of their organization and the geothermal binary power plant. | ・Very close (<1.5 km)  ・Close (1.5-3 km)  ・Middle (3-10 km)  ・Far (10-100 km)  ・Very far (>100 km) |
| Hometown | Their hometown. | ・Tsuchiyu  ・Other (including unknown data) |

Supplementary Table 3 Continued.

| **Variable** | **Explanation** | **Choice** |
| --- | --- | --- |
| Profit/Non-profit | Whether their organizations are profit or non-profit. | ・Profit  ・Non-profit |
| Number of groups | The number of their belonging groups in Tsuchiyu, including their main organizations. | ・Few (1)  ・Middle (2-3)  ・Many (≥4) |
| Environment | The responses for Q19 in Supplementary Table 1. | ・Disagree (1-2)  ・Neutral (3)  ・Agree (4-5)  (1: strongly disagree, 2: disagree, 3: equally agree and disagree, 4: agree, 5: strongly agree) |
| Benefit | The responses for Q24 in Supplementary Table 1. | ・Disagree (1-2)  ・Neutral (3)  ・Agree (4-5)  (1: strongly disagree, 2: disagree, 3: equally agree and disagree, 4: agree, 5: strongly agree) |
| SLO factors | The average response of the questions for each SLO factor. | ・Low (1-2.333)  ・Middle (2.333-3.667)  ・High (3.667-5) |


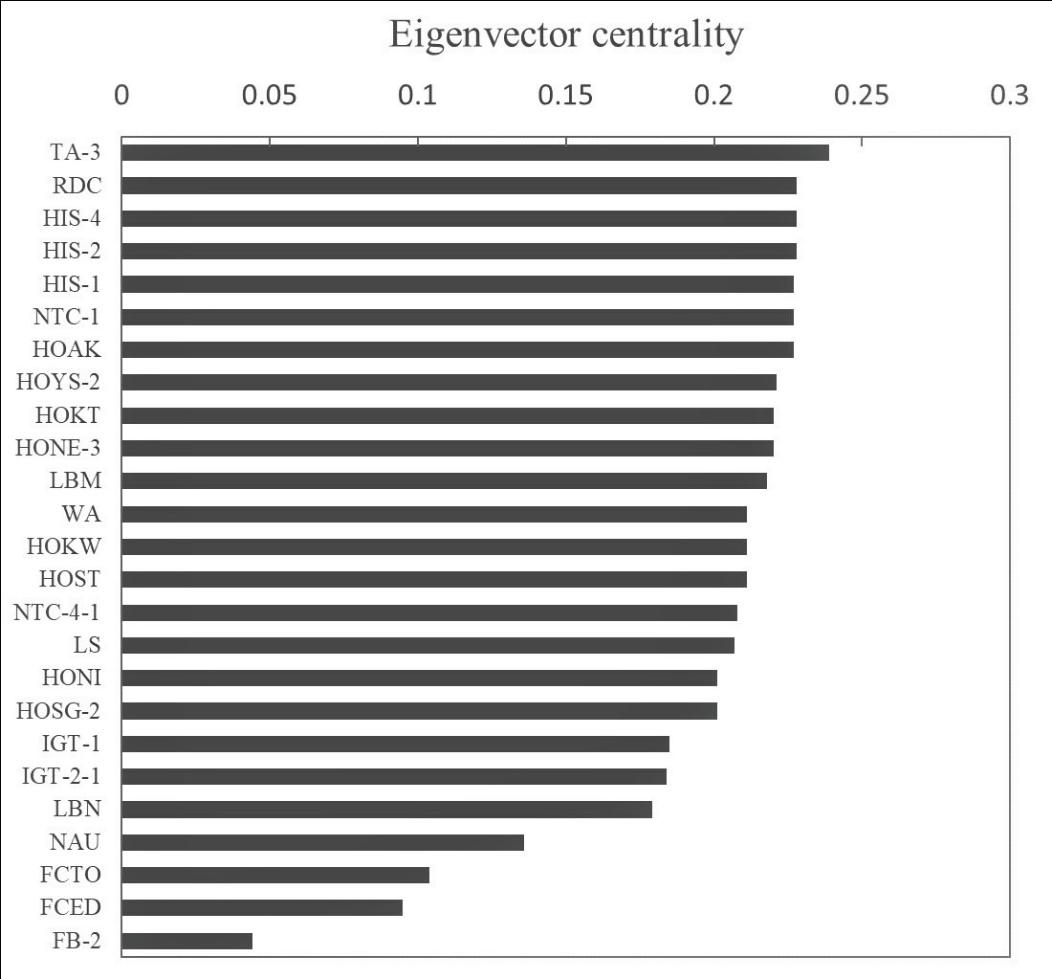


Supplementary Figure 1 Eigenvector centrality of responses from stakeholders in Tsuchiyu.
